# Virtual training leads to real acute physical, cognitive, and neural benefits on healthy adults: study protocol for a randomized controlled trial

**DOI:** 10.1186/s13063-019-3591-1

**Published:** 2019-09-11

**Authors:** Dalila Burin, Noriki Yamaya, Rie Ogitsu, Ryuta Kawashima

**Affiliations:** 1 Smart Aging International Research Center (SAIRC), Tohoku University, 4-1 Seiryocho, Aobaku, Sendai, 980-8575 Japan; 2 Institute of Development, Aging and Cancer (IDAC), Tohoku University, 4-1 Seiryocho, Aobaku, Sendai, 980-8575 Japan

**Keywords:** Executive functions, Stroop task, Functional near-infrared spectroscopy, Prefrontal cortex, Immersive virtual reality, Virtual high-intensity intermittent training, Heart rate, Body ownership, Sense of agency

## Abstract

**Background:**

Keeping a certain level of physical activity has beneficial effects on the body itself but also, surprisingly, on cognition: specifically, physical high-intensity intermittent aerobic exercise (HIE) can show improvement on cognitive executive functions. Although, in some cases performing strength or aerobic training is problematic or not feasible. Immersive virtual reality (IVR) can induce the illusory feeling of ownership and agency over a moving virtual body, therefore showing comparable physiological reactions: for example, if an individual is sitting on a chair but his virtual body climbs a hill, the individual’s heart rate increases coherently, as if he is actually walking.

In this study, we investigate whether this same illusion can show beneficial consequences on the body as well as on executive functions (using the color-word matching Stroop task) and on its neural substrates (using functional near-infrared spectroscopy [fNIRS]).

**Methods:**

In a cross-over randomized controlled trial, 30 healthy young adults will experience HIE training in IVR (i.e. the virtual body will perform eight sets of 30 s of running followed by 30 s of slow walking, while the participant is completely still) according to two random-ordered conditions: during the experimental condition, the virtual body is displayed in first-person perspective (1PP), while in the control condition, the virtual body is displayed in third-person perspective (3PP). To confirm that individuals have the illusion of ownership and agency over the virtual body in 1PP (and not in 3PP), we will record the heart rate, in addition to subjective questionnaires. Before and after every IVR sessions (one week apart), we will measure cortical hemodynamic changes in the participants’ prefrontal cortex using the fNIRS device during the Stroop task’s execution.

**Discussion:**

From a theoretical perspective, we could prove that the sense of body ownership and agency can modulate physical and cognitive parameters, even in the absence of actual movements; from a clinical perspective, these results could be useful to train cognition and body simultaneously, in a completely safe environment.

**Trial registration:**

University Hospital Medical Information Network Clinical Trial Registry, UMIN000034255. Registered on 1 October 2018.

**Electronic supplementary material:**

The online version of this article (10.1186/s13063-019-3591-1) contains supplementary material, which is available to authorized users.

## Background

It is widely known that a constant level of physical exercise is one of the key components for overall health and to postpone aging-related disorders [1–5], in somatic and cognitive terms [6–10]. Surprisingly, several recent studies have shown that aerobic exercise has beneficial consequences not only on bodily but also on cognitive functions [11–16]. Intriguingly, Kujach et al. [17] demonstrated that a high-intensity intermittent exercise (HIE)-based intervention has beneficial immediate effects on the cognitive function of sedentary young adults similar to the benefits of long-term physical adaptations [15, 18]: specifically, the HIE model they proposed consists of eight sets of 30 s of cycling exercise at 60% of the individual’s maximal aerobic power followed by 30 s of rest, for a total of 8 min. Before and after the HIE, they repeated the color-word matching Stroop task (hereinafter, Stroop task) that is widely known to measure executive functions [19–21] and they recorded with a multi-channel functional near-infrared spectroscopy (fNIRS), which allows the monitoring of cortical hemodynamic changes, specifically in the prefrontal cortex (PFC), where executive functions are localized [22, 23]. Significant behavioral improvement in inhibiting cognitive conflict induced by the Stroop-interference effect occurred after HIE and, as a neural counterpart, they found a significant increase in the cortical activation in the left dorsolateral prefrontal cortex (DLPFC) specifically after the HIE session. Therefore, this HIE-based exercise intervention may provide a strategy for improving cognitive and cardiovascular functions at the same time.

Unfortunately, in some cases, it is complicated (e.g. sedentary, very busy people, elderly in a frailty condition), sometimes even impossible (e.g. cardiopathic individuals, patients recovering after long-term disease, patients with motor impairment), to perform constant physical exercise [24] or even a short-term high-impact physical exercise. These lifestyles characterized by the absence of constant physical activity clearly have negative effects on the wellbeing of the body and, as described previously, can limit the improvement of cognitive abilities or even contribute to their decline.

For those situations, a potential solution could be to bypass the actual execution of movements by creating a condition where bodily and cognitive reactions are comparable to real ones. In order to do so, an effective innovative technique seems to be immersive virtual reality (IVR) because it allows showing a virtual world and, more importantly, a virtual body (also called avatar) that is extremely realistic and plausible [25–28]. With the sole use of a visor equipped with two that show the virtual environment [29], it creates a strong feeling of “being there,” just exploiting the visual stimulation [30–37] (different from what happens with other multisensory illusions; see for example [27–29]).

Several recent papers have shown that it is not only the simple display of an avatar in first-person perspective (1PP) that creates the illusory sense of body ownership (SoBO) over the virtual body [38–40], but its movements also create the illusory sense of agency (SoA) over them [32, 33, 41]; the actions performed by their own virtual body are subjectively considered as own generated and they arise subjective, behavioral, and physiological reactions in the real individual’s body perfectly analogously with what happens in the physical world [32, 33]. For example, when the movements of their own virtual body do not match the real ones, an unconscious adjustment of the actual performance occurs, driven by the sense of body ownership over the avatar [41]. But even in the absence of actual movements, if the virtual body moves while the real body is totally still, the illusion can trigger measurable reactions perfectly comparable to the ones during an actual movement execution, online [33, 41] or in terms of consequences [32, 42, 43]: interestingly, for our purposes, it has been shown that if the virtual body, displayed in 1PP, climbs a hill, the real participant’s heart rate (HR) increases, exactly as would happen in the physical world. In this case, it seems to be a physiological counterpart of the SoBO visual illusion [33]. In summary, IVR can provide effective investigating methods to better understand the relationship between SoBo and SoA [44] and can provide potential solutions, complementary to the existing ones, for a clinical population (see [43] for a review).

### Purpose

In this study, we combined the efficacy of the HIE on cognitive functions [17] with the possibility of manipulating SoBO and SoA offered by IVR [33, 41]. Our goal is to demonstrate whether a virtual HIE-based intervention performed exclusively by the considered-as-own virtual body, while the real individuals’ body is totally still (hereinafter vHIE), has acute cognitive and somatic beneficial effects on the real body, comparable to the ones that arise after actual physical training. We will compare the behavioral performance (speed and accuracy) with the Stroop task during the recording of the brain activity of the PFC with fNIRS (as in [15]), before and after the vHIE, in two interventions where we manipulate the visual perspective (1PP vs 3PP), known to induce or disrupt the SoBO and SoA over the avatar.

## Methods/design

**Fig. 1:**
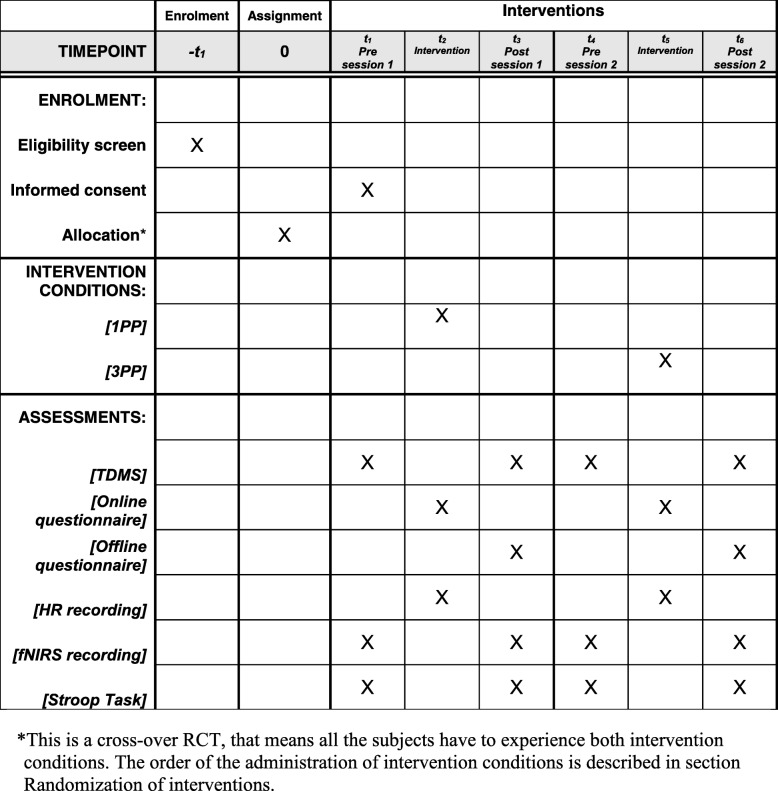
Schedule of enrolment, interventions, and measurements, according to SPIRIT guidelines

**Fig. 2:**
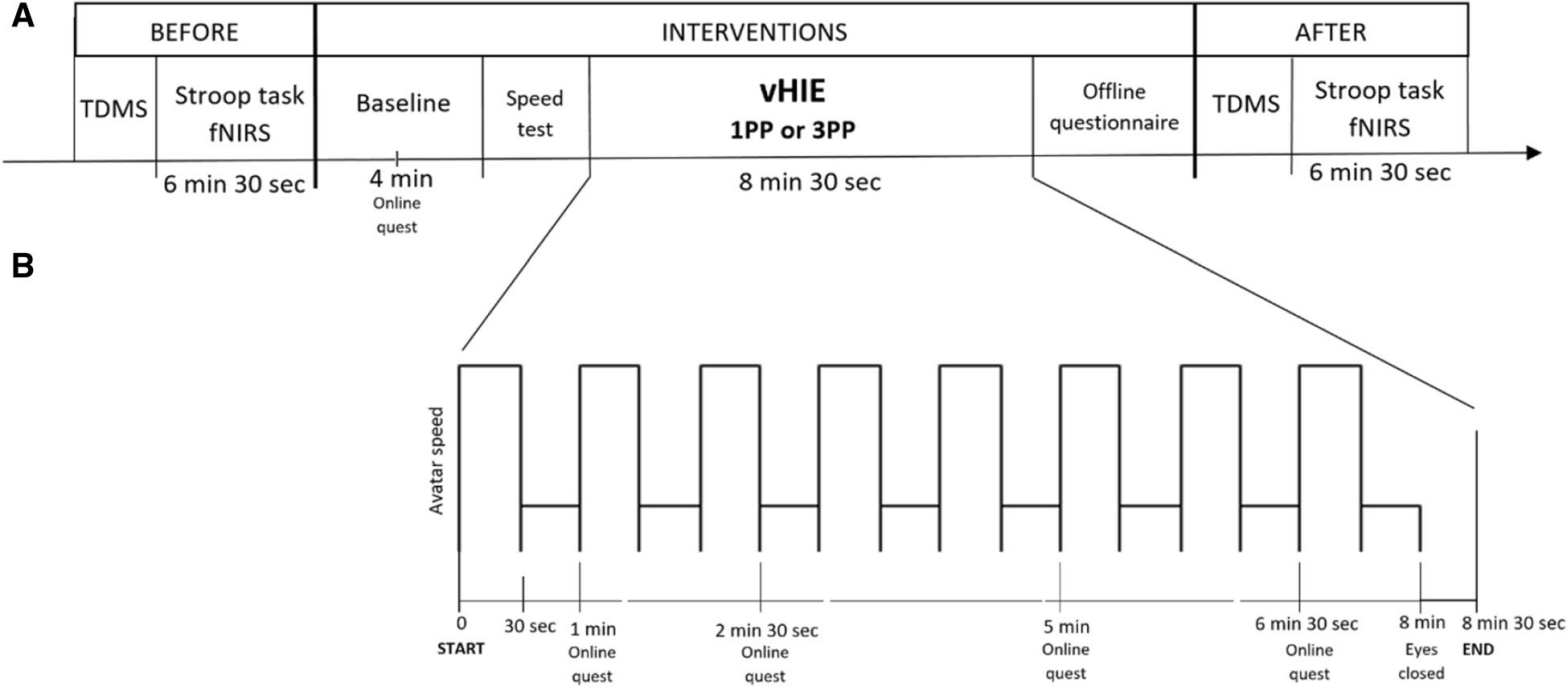
General procedure of the study. **a** The protocol of one experimental session (in both sessions the procedure is the same, the intervention condition changes) with its timeline. The first part describes the measurements before the intervention (TDMS and Stroop task with fNIRS recording); the central part corresponds to the vHIE intervention (that, according to the session, may be in 1PP or 3PP). **b** The intervention details: every 30 s, the speed of the avatar changes from slow walking to running; at specific timepoints, we will administer the online questionnaire for SoBO and SoA. The third part of A. describes the measurements after the intervention (same as before the intervention)

The protocol was developed according to the SPIRIT (see Additional file 1) and CONSORT (see Additional file 2) guidelines for randomized controlled trials (RCT) [45]. The schedule for this study is displayed in Fig. 1, while the general procedure is shown in Fig. 2.

### Trial design

This study is a cross-over (within-subjects) RCT conducted at the Smart-Aging Research Center (Institute of Development, Aging and Cancer, Tohoku University), Sendai City, Miyagi Prefecture, Japan. All participants will perform both experimental and control conditions (in other words, all participants will act as their own controls). This study (version 1.0) was registered with the University Hospital Medical Information Network (UMIN) Clinical Trial Registry (UMIN000034255) on 1 October 2018.

### Participants recruitment and eligibility criteria

Thirty healthy young adults (15 women) will be recruited among students of Tohoku University (Sendai, Japan) via a specific online system. They will be reimbursed 1000 Japanese Yen per hour for their participation.

Participants must be native Japanese-speaking men and women aged 20–35 years and self-report to be right-handed. In addition, they have to report no history of neurological, psychiatric, or motor disorders and normal color vision. To avoid eventual problems during the IVR sessions, we will exclude individuals that report to be extremely sensitive to motion sickness (e.g. nausea while driving a car).

### Randomization of interventions

The two intervention conditions will be administered to all participants in two separate sessions (one week apart). Random assignment to the first intervention using an online program (http://www.graphpad.com/quickcalcs/index.cfm) will take place [43]. Each participant is first assigned to a condition non-randomly (the random number generator is seeded with the time and the day). The assignment of each individual is then swapped twice (to make sure it is really random) with the condition assignment of a randomly chosen participant. Half of the participants (15 individuals) will perform the experimental condition first, while the other half will perform the control condition first. The sequence, and with it the assignment to the first intervention condition, is hidden from the researcher that enrolls the participants until the intervention starts to avoid biases; once the intervention begins, the sequence is no longer blinded from anyone.

### Interventions

Participants will visit the laboratory (located in the Smart-Aging Research Center) for two sessions, one week apart, corresponding to the two intervention conditions. In both sessions, the participants sit comfortably on a stool with their feet firmly resting on the ground and their arms relaxed by their sides. They have to wear the Oculus visor (oculus.com), equipped with two lenses that show the virtual world. They will be instructed to keep their bodies still but they can move and rotate their head, to explore the virtual body and the environment. The virtual environment, as well as the virtual bodies and the animations, is modelled in 3D Studio Max 2015 and implemented in Unity3D: it represents an open space with a green floor (simulating a meadow) and a natural-looking illuminated sky, with a visible horizon. Participants will see a gender-matched life-sized humanoid virtual body.

The intervention will be performed in two conditions. The experimental intervention displays the virtual body in 1PP, where the virtual body substitutes and is spatially coincident with the real one; in other words, to observe the virtual body, the participant has to look towards him/herself. The control intervention consists of displaying the same virtual body in 3PP, where the virtual body is collocated approximately 1.5 m to the left of the real participant’s body; in other words, to observe the virtual body, the participant has to rotate his head and look towards his left side.

In both conditions (1PP and 3PP), the intervention starts with a familiarization phase of 4 min (hereinafter baseline) where the gender-matched virtual body is displayed but there is no animation yet (i.e. the virtual body in both 1PP or 3PP is standing still). This phase is necessary to induce the illusory sensation of ownership over the virtual body (in 1PP) or not to induce it (in 3PP) (see the “Measurements” section for the online questionnaire on SoBO and SoA) for the baseline recording of the HR (see the “Measurements” section) and to check for eventual sickness problems due to the virtual display.

After that, the actual intervention consists of a vHIE performed by the virtual body exclusively: while seated, participants will see the virtual body (either in 1PP or 3PP) alternating 30 s of running and 30 s of slow walking, for a total of 8 min [17, 33]. After that, we will ask the participant to stay still and close his/her eyes, while we still record an extra 30 s of the HR (see the “Measurements” section) (see Fig. 2b). While the slow walking animation will be the same for all participants, in order to choose the speed of the running animation, after the familiarization phase, we will show four different options on the virtual body and individuals verbally report which one is subjectively perceived as considerably fast but feasible. This is necessary to maximize the possibility of having an actual physiological activation but, at the same time, to display an animation that is subjectively plausible so as to not break the illusion of ownership in 1PP.

### Measurements

#### Heart rate

During the interventions, in order to check if participants actually perceive the virtual body as their own (in 1PP and not in 3PP), we will record the HR with a Polar H10 HR monitor, controlled by a specific application via Bluetooth. Participants have to wear an elastic strip around the chest on which the HR monitor is attached, positioning it close to the heart, in order to record the HR for each intervention conditions (1PP and 3PP) during the baseline (4 min), the intervention (4 min running, 4 min slow walking, for a total of 8 min) plus an extra 30 s (where the participant is still with closed eyes) to check for eventual delay in the recording and to wait for the synchronization of the HR.

#### Online and offline questionnaires on SoBO and SoA

We will administer an online questionnaire for the subjective experience of SoBO and SoA. To control for eventual changes in subjective feelings during the intervention at different timepoints (in the middle of the baseline phase [i.e. after 2 min from the beginning if the IVR session], during the intervention at 1 min [i.e. while the avatar is running], at 2.5 min [i.e. while the avatar is slow walking ], at 5 min [i.e. while the avatar is running], and at 6.5 min [i.e. while the avatar is slow walking]), we will ask participants to rate their level of agreement (on a 1–7 Likert scale where 1 means “totally disagree” and 7 means “totally agree”) with respect to four statements: two of them (one is a “real statement” that checks for the actual presence of the illusion, while the other is a “control statement”) about SoBo and the other two about SoA (see Table 1) (see Fig. 2b).

**Table 1:** Online questionnaire on SoBO and SoA

s1	SoBO	I feel as if I’m looking at my own body.
s2	SoBO control	I feel as if the virtual body belongs to another person.
s3	SoA	The virtual body moves just as I want, as if I am controlling it.
s4	SoA control	I feel as if the virtual body is controlling my will.

Questionnaire verbally administered during the vHIE (at 5 timepoints, at 2 min from baseline, and 1 min, 2.30, 5 and 6.30 min of vHIE) concerning the subjective participant’s feelings during the intervention. The statements s1 and s2 concern the SoBO, while the statements s3 and s4 concern the SoA. The statements s1 and s3 are “real statements” while statements s2 and s4 are “control statements.” Individuals will rate their level of agreement to the following statements on a 1–7 Likert scale (1 means “complete disagreement” and 7 means “complete agreement”).

Another questionnaire will be administered right after every IVR session (both after 1PP and 3PP intervention conditions) to check details for feelings of movement, motor control, and physical effort (see Table 2) (see Fig. 2 a). The statements are selected and adapted from previous studies [33, 41].

**Table 2:** Offline questionnaire on SoBO and SoA

s5	Located	During the experiment, I felt as if my body was located where I saw the virtual body to be.
s6	Ownership	During the experiment, I felt that the virtual body was my own body.
s7	Standing	During the experiment, I felt that I was standing upright.
s8	My movements	During the experiment, I felt that the leg movements of the virtual body were my movements.
s9	Agency	During the experiment, I felt that the leg movements of the virtual body were caused by my movements.
s10	Ownership control	During the experiment, I felt that the virtual body belonged to someone else.
s11	Effort	I felt I had to give extra physical effort when the virtual body was walking faster.
s12	Vection	I felt that I was moving through space rather than the world moving past me.
s13	Walking	I felt that I was walking.
s14	Dragged	I felt that I was being dragged.
s16	Sliding	I felt that I was sliding.

Questionnaire self-administered after the vHIE interventions concerning the subjective participant’s feelings during the previous intervention. The underlying explored domains are listed in the first column (not shown to the participant) while the corresponding statements are listed in the second column. Individuals will rate their level of agreement to the following statements on a 1–7 Likert scale (1 means “complete disagreement” and 7 means “complete agreement”).

#### Stroop task

To measure the actual efficacy of the proposed intervention, we will record cortical hemodynamic changes in the participant’s PFC using the functional near-infrared spectroscopy (fNIRS) device before and after every IVR session (either 1PP and 3PP) during the execution of the color-word matching Stroop task (see Fig. 2 a).

We will adopt the Stroop task as previously used in several studies [17, 22, 46–48]. The Stroop task we plan to use consists of 30 trials, including 10 neutrals, 10 congruent, and 10 incongruent, presented in random order. For all trials, two words are displayed on the monitor one above the other: specifically for neutral trials, the upper row consists of XXXX printed in red, white, blue, pink, or yellow, and the lower row shows the words “RED,” “WHITE,” “BLUE,” “PINK,” or “YELLOW” printed in black. For congruent trials, the upper row contains the words “RED,” “WHITE,” “BLUE,” “PINK,” or “YELLOW” printed in the congruent color (e.g. RED was printed in red) and the lower row contains the same color words printed in black. For incongruent trials, the color word in the upper row is printed in an incongruent color (e.g. RED was printed in yellow) to produce cognitive interference between the color word and the color name (i.e. Stroop interference). All words were written in Japanese (hiragana). The lower row is presented 100 ms later than the upper row, in order to achieve sequential visual attention. Between each trial a fixation cross is shown, as an inter-stimulus interval, for 9–13 s to avoid timing prediction [17, 22, 49]. The stimulus remains on the screen for 2 s, independently from the participant’s answer. We will train participants to decide whether the color of the upper word (or letters) corresponds to the color name of the lower word by pressing button 1 on the keypad to give “yes” responses or button 2 to give “no” responses with their forefingers. Of the presented stimuli, 50% were correct (the correct answer is “yes”). We recorded response time (RT) and error rate (ER) as variable.

The Stroop task will be administered entirely via computer (to avoid biases due to presence of the researcher, especially concerning RT); for the present study, the Stroop task has been implemented using E-prime 2.0.

#### Functional near-infrared spectroscopy

**Fig. 3:**
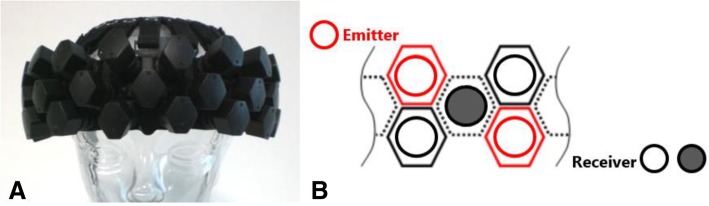
The fNIRS system used in this study. **a** The headset of the fNIRS is shown on a mannequin. **b** Graphic representation of the detail and distribution of the capsules on the headset. Source: http://neu-brains.site/ [50]. Permission to reproduce the figure granted by NeU Corporation, Japan

We will use a wearable (without optical fibers) fNIRS optical topography system (WOT-HS, Hitachi Corporation & NeU Corporation, Japan). The NIRS headset (Fig. 3a) sends the signals to the Wearable Optical Topography High Sensitivity software Version 1.04 (Hitachi Solutions, Inc.) through a Control Box.

This system is provided with 35 capsules, placed 3 cm away from each other, into which microprocessors, NIR emitting or high-sensitivity receiving sensors are packaged: the top and the bottom lines of the capsules alternate emitting and receiving sensors, while the central line comprises receivers only (Fig. 3 b); this new multi-distance measurement mode, including a total of 12 emitting and 23 receiving (11 of them are short-distance receivers) sensors, significantly reduces the biological noise on the hardware side, resulting in 34 channels over lateral and anterior PFC. The device will be positioned on the forehead by centering the specific mark on bottom line of probes at the Fpz (10% of the distance between Nasion and Inion), according to the international 10–20 system [51].

The device detects the concentration of oxygenated hemoglobin (O2Hb), deoxygenated hemoglobin (HHb), and total hemoglobin, calculated in units of millimolar-millimeter (mM·mm) [52], by applying two short-distance wavelengths of NIR light (850 nm, 730 nm) to monitor the above mentioned cortical hemodynamic changes in the PFC during the Stroop task [53].

#### Two-Dimensional Mood Scale

The Two-Dimensional Mood Scale (TDMS) is an effective measure to record changes in psychological mood states. The TDMS was developed as a psychometric scale using eight mood-expressing items (energetic, lively, lethargic, listless, relaxed, calm, irritated, and nervous) that, when combined, express mood states of pleasure and arousal [54]. We will ask participants to rate their present psychological state using a six-point Likert scale from 0 = “Not at all” to 5 = “Extremely” [17]. They will repeat the TDMS right before the pre-Stroop task (before the vHIE), as well as right after the post-Stroop task (after the vHIE) for each session (1PP and 3PP) (see Fig. 2a).

### Outcomes

#### Primary outcomes

Considering that the main goal of this study is to determine that vHIE intervention performed with the own virtual body (1PP) has a beneficial effect on cognitive executive functions, the primary outcome of this study is the Stroop task (i.e. the cognitive domain), specifically the two related measurements (RT primarily and ER secondarily). The Stroop task is considered the primary outcome here because it is widely known as a measure for executive brain functions [21, 55]. As a key effect of the Stroop task, we will consider the so-called “Stroop interference” as the (average of incongruent trials– average of neutral trials) contrast, which is assumed to represent Stroop interference, for both RT and ER in each condition (1PP and 3PP).

Specific timepoints (before and after the vHIE) will be considered, but the main comparison in order to find a significant effect of vHIE on cognitive functions will be 1PP versus 3PP.

#### Secondary outcomes

Secondary outcomes are the data extracted from the fNIRS recording, as a neural counterpart of the behavioral measurements. We will focus specifically on O2Hb changes before and after the vHIE in the two conditions (1PP and 3PP).

In addition, we will examine the relation between Stroop task and the induced cortical activation, by means of a correlation between behavioral data from the Stroop task (RT and ER) and optical data from fNIRS. For each trial of the Stroop task (neutral, congruent, and incongruent), we will average the O2Hb data for 2 s before, 2 s during, and 10 s after the presentation of the stimulus (expecting a peak at 4–11 s after the display); also in this case, we will obtain the Stroop interference by subtracting (incongruent-neutral) O2Hb data. As for the other outcomes, the main comparison to test our hypothesis will be between 1PP and 3PP.

#### Additional outcomes

Additional multiple outcomes are the results obtained during the intervention itself (i.e. HR, online and offline questionnaires), in addition to the TDMS scale results.

### Sample size

Considering that the main outcome of this study is the RT of the Stroop task (which is claimed by many to be one of the indicators of executive performance), which typically has a moderate effect, we calculated a sample size of 28 individuals (with α error probability set at 0.05 and power set at 0.8). We decided to recruit 30 participants to eventually control for drop-off.

### Data management

Every participant will be assigned a code according to their arrival at the laboratory to attend the first session, independently from the intervention condition (S01, S02, etc.). Personal information and data for this experiment are handled exclusively by the involved researchers (Tohoku University). According to the institutional policy [56], in order to ensure the security of all data and personal information, it is limited to the involved researchers (and to external parts only after their formal approval). After the end of the experimental period, any information directly linking data back to the participant will be discarded to guarantee anonymity. Personal data eventually collected will not be shared or disclosed in any way.

### Statistical analysis

The current RCT is designed to determine whether a virtual intervention has a beneficial effect on executive functions; this is possible only if some assumptions, during the intervention itself, are satisfied (e.g. embodiment over the virtual body in 1PP). Consequently, the data analysis will be organized in two phases: in the first phase, the results collected during the intervention itself will be checked (i.e. HR, online and offline questionnaires) in order to see whether the virtual intervention is actually effective. In the second phase, the measurements will be analyzed to check the hypothesis (i.e. Stroop task and fNIRS data) and their correlations.

#### Heart rate data

For the HR, the data of the three recording periods (baseline, running, and slow walking) for 4 min each will be averaged: the averaged baseline (HRb) results will be subtracted by the averaged running (HRr) and the averaged walking (HRw) results:

$$ \mathrm{dHRr}=\left(\mathrm{HRr}-\mathrm{HRb}\right);\mathrm{dHRw}=\left(\mathrm{HRw}-\mathrm{HRb}\right) $$

Then, an ANOVA 2 × 2 with factor HR speed at two levels (dHRr = fast, dHRw = slow) and factor CONDITION at two levels corresponding to the intervention conditions (1PP, 3PP) will be run. We predict finding a significant increase in the dHRr in the 1PP condition, with respect to all the other measurements, in order to confirm the physiological counterpart of the embodiment of the virtual body in 1PP only.

#### Data from the online and offline questionnaires on SoBO and SoA

As a subjective counterpart, the data from the questionnaires will be analyzed. The online questionnaire will be repeated in five different timepoints (baseline, 1 min, 2.30 min, 5 min, and 6.30 min): to exclude temporal effect, we will first compare the factors TIME (corresponding to the previously mentioned timepoints where the online questionnaire is administered) and QUESTION (corresponding to the four statements of the online questionnaire) with an ANOVA 5 × 4. We can eventually proceed to an average of the statements across timepoints and then we will compare the four statements (specifically comparing results from “real statements” with “control ones”) and the CONDITION (1PP, 3PP) in an ANOVA 4 × 2. We predict finding higher levels of ownership and agency for the real statements in 1PP, in respect to the control statements and in respect to 3PP.

Concerning the offline questionnaire (administered at the end of every IVR condition), we will analyze the data comparing statements among the two conditions (1PP and 3PP).

#### Stroop task’s RT and ER

The second phase of the data analysis concerns the data collected before and after every IVR intervention, i.e. the results necessary to confirm our main hypothesis (the efficacy of IVR training on cognition).

As previously mentioned, in the Stroop task we will include two measurements, RT and ER: the (incongruent – neutral) contrast, which is assumed to represent Stroop interference, will be calculated. Both (RT and ER) will be analyzed by means of a repeated-measures ANOVA with TIME (before, after) and CONDITION (1PP, 3PP) as within-subject factors. As supplementary analysis, we will check for eventual differences between the two sessions considering only the Stroop task before the vHIE (predicting not differences). According to our hypothesis, we predict finding a shorter RT and lower ER after the vHIE in 1PP compared to 3PP.

#### fNIRS data

The optical data from fNIRS will be analyzed based on the modified Beer-Lambert law [57]. We will set the sampling rate at 10 Hz and analyze the difference between O2Hb and HHb signals.

We will employ the general linear model to identify O2Hb and HHb hemodynamic brain responses with reference to experimental factors. If necessary, we will combine few adjacent channels in order to create region of interest (ROI) [58] according to the LBPA40 anatomical labelling system [59].

Then, the changes in the concentration of O2Hb and HHb for each channel will be treated according to the following steps:

1. Excluding skin blood flow (i.e. heartbeat pulsations) from raw data by using the specific software provided.
2. Pre-processing each channel using 0.01–0.5 Hz bound pass filter to account for the effects of Mayer waves, high-frequency fluctuations, and baseline drift [60].
3. Performing a 3-s moving average to smooth the raw O2Hb concentrations [61, 62].
4. We will use the color-word matching Stroop task in an event-related design which presents each neutral, congruent, and incongruent conditions in random order, so we will pick up the changes related to each condition in the concentration of O2Hb from each channel (or the combined ROIs). In particular, we will calculate the mean of the changes in the concentration of O2Hb 2 s before onset task, as a “baseline,” and 10 s after the onset of the inter-stimulus interval as “vascular response” in each Stroop task condition [46]. That will be necessary because NIRS signals are delayed with respect to participants response [46, 63].
5. Translating the mean of O2Hb concentrations of each channel to normalized values using linear transformations, so that the mean ± standard deviation of O2Hb level in the 2 s of the baseline period is 0 ± 1 (AU). This method will be useful because of the avoidance of the influence of differential pathlength factors among the individuals and that of cortical regions [60, 64, 65].
6. Lastly, the channels over target ROI areas, eventually, will be averaged in each Stroop task condition respectively.

#### TDMS data

We will calculate levels of arousal and pleasure from TDMS scores. At first, we will confirm that the sample represents the normal population, i.e. shows a normal distribution (all *p* values for arousal and pleasure level in both intervention conditions have to be > 0.05 at Shapiro-Wilk test). In addition, we will check if the levels of arousal and pleasure are not different between sessions (comparing results before 1PP and before 3PP) so that all participants start from the same baseline level. That means we can proceed to subtract the results after the intervention from the results before the intervention (after-before).

By performing an ANOVA comparing 1PP and 3PP, we predict finding an increased level of arousal (but not necessarily of pleasure) after the vHIE, especially after the intervention in 1PP, as described in previous studies [17].

#### Correlations

The crucial correlation for our hypothesis concerns the relationship between Stroop task and fNIRS data: we will first investigate the cortical regions mainly activated during the Stroop task before the vHIE in both intervention conditions, as a baseline reference. Then, the (incongruent – neutral) contrasts for the two intervention conditions will be averaged as substrates for ROI analysis. The (incongruent – neutral) contrast for RT and ER with O2Hb changes in all ROIs will be treated with a repeated-measures ANOVA considering as factors intervention condition (1PP and 3PP) and time (before and after the vHIE).

In addition to that, as described in previous studies, we will examine the relation between Stroop data and cortical activation or TDMS results in a binominal manner [22, 49]: for each variable the following contrast will be calculated {[(incongruent – neutral) of after vHIE] – [(incongruent – neutral) of before vHIE] in 1PP condition} – {[(incongruent – neutral) of after vHIE] – [(incongruent – neutral) of before vHIE] in 3PP condition}. Both values will be subjected to the McNemar test to examine the correspondence between the two incidences [66].

### Data monitoring and auditing

According to the recommended guidelines for clinical research in Japan (https://www.mhlw.go.jp/topics/bukyoku/seisaku/kojin/dl/161228rinsyou.pdf) and the institution regulations, data monitoring by a third party is not applicable for the RCT proposed here, since we are not providing participants with any medications nor surgery [56].

### Risks and benefits to participants

Participants are unlikely to encounter any serious risks or burdens.

Participants will possibly experience fatigue and discomfort during the Stroop task and the fNIRS recording. The participant will be informed in advance that, should they feel any discomfort, the test can be interrupted at any time.

The interventions in IVR are not particularly difficult and should not cause the participants any pain since they just have to stay still. Possibly, participants may experience a sense of dizziness or nausea due to the IVR display; to avoid that, exclusion criteria mention that people who are very sensitive to motion sickness are excluded. If, for some reason, the participant experiences any discomfort, they will have been previously informed that they may immediately interrupt the training. Any adverse events will be formally reported.

In accordance with the regulations of the institution, the participants will be given a monetary reward based on the number of hours invested performing the experiment. Therefore, in case the participant decides to leave the ongoing experiment, he/she will not receive any reward (i.e. only participants who complete the entire experiment will be rewarded).

## Discussion

The background of this RCT protocol study starts with two assumptions, based on data presented in the literature: (1) HIE has acute beneficial consequences on executive functions and their neural basis [17, 67]; and (2) the movements of their own virtual body can generate measurable consequences on their real body, comparable to the ones when we actually move [33, 41].

Consequently, we hypothesized that the same HIE intervention performed by their own virtual body (and not by the real participants’ bodies) can have the same beneficial acute effects on executive functions.

In order to confirm our hypothesis, 30 healthy young adults will undergo a vHIE, where the virtual body exclusively (the real body is still sitting) will perform 8 min of training, alternating 30 s of running and 30 s of slow walking; the same vHIE will be performed in two intervention conditions: 1PP, the virtual body will be displayed with the real one, spatially replacing it, and so it will be considered as their own; in 3PP, the virtual body will be displayed 1.5 m away on the right side of the real one, and so it will simply be considered as someone else. During the vHIE, we will record the HR: we predict finding a fluctuating trend of the HR during the intervention in 1PP (and not in 3PP), coherent with the virtual movements, as if the individual is actually performing them. In addition, we will ask the participants to report verbally any subjective feelings during the intervention, predicting that increased sensations of SoBO and SoA in 1PP respect to 3PP will be found.

To test our main hypothesis about acute beneficial effects on executive functions, before and after the two vHIE intervention conditions, we will record the participants’ performance of the Stroop task; at the same time, we will record cortical hemodynamic changes over the PFC with the fNIRS. We predict finding better performance, in terms of reaction time and accuracy, in the Stroop task and higher activation of the neural correlated areas (i.e. PFC) after the vHIE in 1PP (and not after 3PP).

If we confirm our hypothesis, it means that virtual training performed only with the own virtual body can raise the same physical but especially cognitive and neural consequences of a “real” physical one.

These results would contribute on different levels: from a theoretical perspective, this study would be another proof that the illusion of ownership over the virtual body is extremely effective and can be manipulated in order to arise effects on different levels (perhaps motor and cognitive). The simple fact that it is possible to show some effects consequent to the virtual illusion, comparable to the real ones, means the virtual movements are as effective as the actual ones. Our idea is that the SoBO (here the main variable manipulated through the perspective) can drive other aspects, such as motor function, and those, in turn, can drive others, such as cognitive functions. Theoretical models of motor control should clearly state the body and the sense of body ownership as a variable that can significantly affect the resulted action [68, 69].

From a clinical perspective, these results will have interesting applications: as previously mentioned, the intervention presented here can be very useful for sedentary people or those suffering from cardiopathy to start physical activity in a totally safe and entertaining way, or combining cardio or power exercise, and the IVR training, maximizing the efficacy of both on somatic and cognitive terms. In more extreme situations, patients with motor disorders can perform the virtual training but with the same beneficial effects as physical training [70].

However, this study has some limitations. All participants have to repeat the Stroop task four times (before and after the two IVR sessions). This could have a learning effect, or an effect due to the repetition itself, as a negative consequence. To avoid this, we will randomize the order in which the intervention conditions will be administered, but it is still possible to have a repetition effect. Another potential limitation concerns the control conditions of the intervention: several previous studies using IVR exploit the 3PP as a control condition. In some cases, it seems to have a sort of effect, although the reported effect is smaller than the one of the experimental condition. Finally, a difference between the previous study with a similar design and the current one concerns the type of intervention: in Kujach et al. [17], the training consists of a cycling exercise, while in our study we decided on a running exercise in order to be as consistent as possible with other previous studies with similar IVR setup [33].

### Trial status

Recruitment of participants started in February 2019 and will end in May 2019.

## Additional files

Additional file 1: SPIRIT checklist. A table specifying where any SPIRIT item has been addressed in the protocol manuscript. (DOC 123 kb)

Additional file 2: CONSORT checklist. A table specifying where any CONSORT item has been addressed in the protocol manuscript. (DOC 217 kb)

## Data Availability

An anonymized version of the main outcome data will be eventually available after publication of the main results paper if explicitly requested.

## Abbreviations

1PP: First-person perspective
3PP: Third-person perspective
Ch: Channels
DLPFC: Dorsolateral prefrontal cortex
fNIRS: Functional near-infrared spectroscopy
HHb: Deoxygenated hemoglobin
HIE: High-intensity intermittent exercise
HR: Heart rate
IVR: Immersive virtual reality
O2Hb: Oxygenated hemoglobin
PFC: Prefrontal cortex
RCT: Randomized controlled trial
ROI: Region of interest
SoA: Sense of agency
SoBO: Sense of body ownership
TDMS: Two-dimensional mood scale
vHIE: Virtual high-intensity intermittent exercise
VR: Virtual reality
